# The Vitamin D Analogue ED71 but Not 1,25(OH)_2_D_3_ Targets HIF1α Protein in Osteoclasts

**DOI:** 10.1371/journal.pone.0111845

**Published:** 2014-11-06

**Authors:** Yuiko Sato, Yoshiteru Miyauchi, Shigeyuki Yoshida, Mayu Morita, Tami Kobayashi, Hiroya Kanagawa, Eri Katsuyama, Atsuhiro Fujie, Wu Hao, Toshimi Tando, Ryuichi Watanabe, Kana Miyamoto, Hideo Morioka, Morio Matsumoto, Yoshiaki Toyama, Takeshi Miyamoto

**Affiliations:** 1 Department of Orthopedic Surgery, Keio University School of Medicine, Shinjuku-ku, Tokyo, Japan; 2 Department of Musculoskeletal Reconstruction and Regeneration Surgery, Keio University School of Medicine, Shinjuku-ku, Tokyo, Japan; 3 Department of Dentistry and Oral Surgery, Keio University School of Medicine, Shinjuku-ku, Tokyo, Japan; 4 Department of Integrated Bone Metabolism and Immunology, Keio University School of Medicine, Shinjuku-ku, Tokyo, Japan; Oklahoma State University, United States of America

## Abstract

Although both an active form of the vitamin D metabolite, 1,25(OH)_2_D_3_, and the vitamin D analogue, ED71 have been used to treat osteoporosis, anti-bone resorbing activity is reportedly seen only in ED71- but not in 1,25(OH)_2_D_3_ -treated patients. In addition, how ED71 inhibits osteoclast activity in patients has not been fully characterized. Recently, HIF1α expression in osteoclasts was demonstrated to be required for development of post-menopausal osteoporosis. Here we show that ED71 but not 1,25(OH)_2_D_3_, suppress HIF1α protein expression in osteoclasts *in vitro*. We found that 1,25(OH)_2_D_3_ or ED71 function in osteoclasts requires the vitamin D receptor (VDR). ED71 was significantly less effective in inhibiting M-CSF and RANKL-stimulated osteoclastogenesis than was 1,25(OH)_2_D_3_ *in vitro*. Downregulation of c-Fos protein and induction of *Ifnβ* mRNA in osteoclasts, both of which reportedly block osteoclastogenesis induced by 1,25(OH)_2_D_3_ *in vitro*, were both significantly higher following treatment with 1,25(OH)_2_D_3_ than with ED71. Thus, suppression of HIF1α protein activity in osteoclasts *in vitro*, which is more efficiently achieved by ED71 rather than by 1,25(OH)_2_D_3_, could be a reliable read-out in either developing or screening reagents targeting osteoporosis.

## Introduction

A cause for concern in developed countries is the increasing number of osteoporosis patients and individuals suffering fragility fractures due to osteoporosis [1]. Estrogen-deficiency due to menopause is a risk factor for both [2]. Vitamin D insufficiency is also reportedly observed in osteoporosis patients with fragility fractures and considered a cause of osteoporotic fractures [3]. Indeed, vitamin D is known to play a crucial role in skeletal development, and lack of the vitamin D receptor (VDR) or low vitamin D intake results in Rickets [4] [5].

Currently, active vitamin D analogues are used in several countries to treat patients with bone and mineral disorders associated with chronic renal disease or osteoporosis [6]. Interestingly, 1,25(OH)_2_D_3_ has been demonstrated to promote osteoclastogenesis in co-cultures of osteoclast progenitor cells and osteoblastic cells [7]; in addition, 1,25(OH)_2_D_3_ elevated receptor activator of nuclear factor kappa B ligand (RANKL), an essential cytokine for osteoclastogenesis, but inhibited expression of OPG, a decoy receptor of RANKL, in osteoblastic cells to promote osteoclast differentiation [8] [9]. In contrast, 1,25(OH)_2_D_3_ was shown to inhibit osteoclast differentiation in osteoblastic cell-free culture systems: osteoclast formation induced by macrophage colony stimulating factor (M-CSF) and RANKL was inhibited in the presence of 1,25(OH)_2_D_3_ [10] [11]. c-Fos protein, an essential transcription factor for osteoclast differentiation, or interferon beta (Ifnβ), an inhibitor of osteoclastogenesis, was downregulated or elevated by 1,25(OH)_2_D_3_, respectively, in osteoclast progenitor cells [10] [11]. However, patients treated with a 1,25(OH)_2_D_3_ pro-drug, alfacalcidol, did not show inhibition of osteoclastic activity or increased bone mass, while patients treated with the vitamin D analogue ED71 exhibited significantly reduced osteoclast activities and increased bone mass [12].

Since postmenopausal osteoporosis is caused in part by estrogen-deficiency, treating of patients with estrogen is one option. However, continuous estrogen administration is associated with adverse effects such as uterine or mammary gland tumors or cardio-vascular disease [13]. Recently, we reported that hypoxia inducible factor 1 alpha (HIF1α) is required for osteoclast activation following estrogen-deficiency and for development of postmenopausal osteoporosis in animal models [14]. We found that in pre-menopausal mice, HIF1α activity in osteoclasts is continuously suppressed by estrogen but then HIF1α accumulate in osteoclasts following estrogen deficiency due to menopause, which in turn activates osteoclastic activity and promotes bone loss. Osteoclast specific HIF1α knockout or administration of a HIF1α inhibitor completely abrogated ovariectomy (OVX)-induced osteoclast activation and bone loss [14]. This study suggests that HIF1α could be a therapeutic target for postmenopausal osteoporosis.

Here, we show that HIF1α is a target of ED71 *in vitro*. HIF1α in osteoclasts was suppressed by ED71 but not by 1,25(OH)_2_D_3_. Since inhibition of osteoclast activity was seen in the patients treated with ED71 but not with 1,25(OH)_2_D_3_, this work confirms that HIF1α could be a target to treat postmenopausal osteoporosis patients.

## Materials and Methods

### Mice

C57BL/6 background wild-type mice were purchased from Sankyo Labo Service (Tokyo, Japan). VDR-deficient mice were established previously [4]. Animals were maintained under specific pathogen-free conditions in animal facilities certified by the Keio University School of Medicine animal care committee. All animal procedures were approved by the Keio University School of Medicine animal care committee.

### Cell culture

To assess *in vitro* osteoclast formation, bone marrow cells isolated from *Hif^flox/flox^* or Ctsk Cre/*Hif^flox/flox^* mouse femurs and tibias were cultured for 72 hours in αMEM (Sigma-Aldrich Co., St. Louis, MO, USA) containing 10% heat-inactivated fetal bovine serum (FBS, JRH Biosciences Lenexa, KS, USA) and GlutaMax (Invitrogen Corp., Carlsbad, CA, USA) supplemented with M-CSF (50 ng/ml, Kyowa Hakko Kirin Co. Tokyo, Japan). Subsequently, adherent cells were collected and cultured under indicated conditions containing M-CSF (50 ng/ml), recombinant soluble RANKL (25 ng/ml, PeproTech Ltd., Rocky Hill, NJ, USA) using 1×10^5^ cells per well in 96-well plates. Osteoclastogenesis was evaluated by TRAP staining [15] [16]. Raw264.7 cells were maintained in DMEM (Sigma-Aldrich Co.) containing 10% heat-inactivated FBS (JRH Biosciences) and GlutaMax (Invitrogen Corp.). For chemical treatment, cells were cultured in phenol red-free media containing 10% charcoal-stripped FBS (Thermo Fisher Scientific K.K., Yokohama, Japan), and treated with 1,25(OH)_2_D_3_ (Wako Pure Chemicals Industries, Osaka, Japan, 10^−7^ M) or ED71 (provided by Chugai Pharmaceutical Co., Ltd, Tokyo, Japan, 10^−7^ M). Hypoxic cultures was performed at 5% O_2_/5% CO_2_ using an INVIVO2 hypoxia workstation (Ruskin Technology Ltd., Bridgend, UK) according to manufacturer's instruction.

### Quantitative PCR analysis

Total RNA was isolated from bone marrow cultures using an RNeasy mini kit (Qiagen), and cDNA synthesis was done by using oligo (dT) primers and reverse transcriptase (Wako Pure Chemicals Industries). Quantitative PCR was performed using SYBR Premix ExTaq II reagent and a DICE Thermal cycler (Takara Bio Inc., Shiga, Japan), according to the manufacturer's instructions. *β-actin* (*Actb*) expression served as an internal control. Primers for *Nfatc1*, *Ctsk*, *DC-STAMP*, *Ifnβ* and *Actb* were as follows.

*Ctsk*-forward: 5′-ACGGAGGCATTGACTCTGAAGATG-3′

*Ctsk*-reverse: 5′-GGAAGCACCAACGAGAGGAGAAAT-3′

*NFATc1*-forward: 5′-CAAGTCTCACCACAGGGCTCACTA-3′

*NFATc1*-reverse: 5′-GCGTGAGAGGTTCATTCTCCAAGT-3′

*DC-STAMP*-forward: 5′-TCCTCCATGAACAAACAGTTCCAA-3′

*DC-STAMP*-reverse: 5′-AGACGTGGTTTAGGAATGCAGCTC-3′

*Ifnβ*-forward: 5′– AAAGCAAGAGGAAAGATTGACGTG -3′

*Ifnβ*-reverse: 5′– ATCCAGGCGTAGCTGTTGTACTTC -3′

*Blimp1*-forward: 5′-TTCTTGTGTGGTATTGTCGGGACTT-3′

*Blimp1*-reverse: 5′-TTGGGGACACTCTTTGGGTAGAGTT-3′

*Irf8*-forward: 5′-CAGGATTACAATCAGGAGGTGGA-3′

*Irf8*-reverse: 5′-TCAAAATCTGGGCTCTTGTTCAG-3′

*β-actin*-forward: 5′-TGAGAGGGAAATCGTGCGTGAC-3′

*β-actin*-reverse: 5′-AAGAAGGAAGGCTGGAAAAGAG-3′

### Immunoblotting

Whole cell lysates were prepared using RIPA buffer (1% Triton X-100, 1% sodium deoxycholate, 0.1% SDS, 150 mM NaCl, 5 mM EDTA, 1 mM dithiothreitol, 10 mM Tris-HCl, pH 7.5) supplemented with a protease inhibitor cocktail (Sigma-Aldrich Co.) and MG-132 (EMD Millipore Corporation). The insoluble fraction was removed by centrifugation followed. Equivalent amounts of protein were separated by SDS-PAGE and transferred to a PVDF membrane (EMD Millipore Corporation). Proteins were detected using the following antibodies: anti-Fos (Santa Cruz Biotechnology, Santa Cruz, CA, USA), anti-HIF1α (Novus Biologicals, Littleton, CO, USA), anti-Actin(Sigma-Aldrich Co.), and anti-Vinculin (Sigma-Aldrich Co.) as previously described [14].

### VDR knockdown

Raw264.7 cells transduced with MISSION shRNA lentiviruses targeting the VDR or with lentiviruses harboring non-target control constructs (Sigma-Aldrich Co.) were generated according to the manufacturer's instructions.

### Statistical analyses

Statistical analyses were performed using an unpaired two-tailed Student's *t-*test (*P<0.05; **P<0.01; ***P<0.005; NS, not significant, throughout the paper). All data are expressed as the mean ± SD.

## Results

### 1,25(OH)_2_D_3_ inhibits osteoclastogenesis more potently than does ED71 *in vitro*

**Figure 1:**
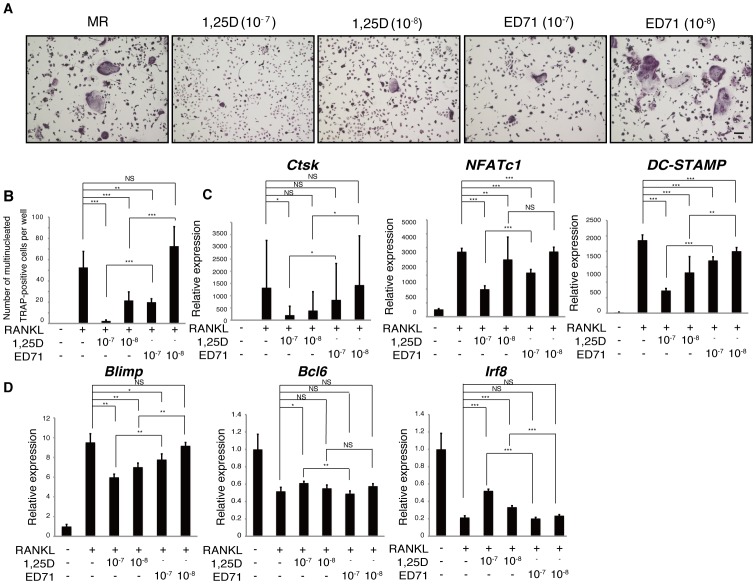
1,25(OH)_2_D_3_ is a more potent inhibitor of osteoclastogenesis *in vitro* than is ED71. (**A, B** and **C**) M-CSF-dependent osteoclast progenitor cells were isolated from wild-type mice and cultured in the presence of M-CSF (M, 50 ng/ml) + RANKL (R, 25 ng/ml) with or without indicated concentrations of ED71 or 1,25(OH)_2_D_3_ (1,25D) for 5 days. Cells were then stained with TRAP (**A**) and the number of multi-nuclear TRAP-positive cells was counted (**B**). Expression of *Ctsk*, *NFATc1* and *DC-STAMP,* all of which are osteoclastic genes, was analyzed by realtime PCR (**C**). Expression of *Blimp1*, *Bcl6* and *Irf8* was analyzed by realtime PCR (**D**). Data represent mean expression of each relative to *Actb* ± SD (*n* = 5). *P<0.05; **P<0.01; ***P<0.001; NS, not significant.

Since treatment with ED71, a vitamin D3 analogue, inhibits osteoclast activity and increases bone mineral density more effectively than does the pro-1,25(OH)_2_D_3_ agent, alfacalcidol [12], we asked whether ED71 inhibited osteoclastogenesis more effectively than 1,25(OH)_2_D_3_ (1,25D) *in vitro* (Fig. 1). To do so, we isolated osteoclast progenitor cells from wild-type mice and cultured them in the presence of M-CSF and RANKL with or without ED71 or 1,25(OH)_2_D_3_. We then evaluated osteoclastogenesis by counting multi-nuclear TRAP-positive osteoclasts and examining expression of osteoclastic genes (Fig. 1A-D). Indeed ED71 significantly inhibited osteoclast differentiation based on both TRAP and gene expression analysis, while 1,25(OH)_2_D_3_ was more effective in inhibiting osteoclastogenesis than was ED71 *in vitro* (Fig. 1A and B). Expression of osteoclast differentiation markers such as *Cathepsin K* (*Ctsk*), *nuclear factor of activated T cells 1* (*NFATc1*) and *dendritic cell specific transmembrane protein* (*DC-STAMP*) was more significantly inhibited by 1,25(OH)_2_D_3_ than by ED71 treatment (Fig. 1C). Induction of B lymphocyte-induced maturation protein 1 (Blimp1) followed by suppression of B cell lymphoma 6 and interferon regulatory factor 8 (Irf8) is reportedly required for osteoclastogenesis [14], [17], [18]. We found that treatment of osteoclast progenitors with 1,25(OH)_2_D_3_ elicited more robust inhibition of *Blimp1* and activation of *Bcl6* and *Irf8* than did treatment with ED71 (Fig. 1D), suggesting that 1,25(OH)_2_D_3_ is more potent in inhibiting osteoclastogenesis induced by M-CSF and RANKL than ED71.

**Figure 2:**
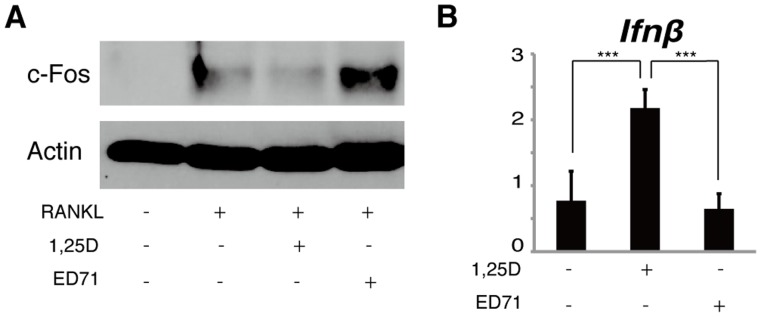
1,25(OH)_2_D_3_ is more active in promoting c-Fos protein inhibition and *Ifnβ*-induction in osteoclasts compared with ED71. (A and B) M-CSF-dependent osteoclast progenitor cells were isolated from wild-type mice and cultured in the presence of M-CSF alone (50 ng/ml) or M-CSF + RANKL (25 ng/ml) with or without 10^−7^ M ED71 or 1,25(OH)_2_D_3_ (1,25D) for 5 days. c-Fos protein was then assessed by western blot (A). *Ifnβ* expression was analyzed by realtime PCR (B). Data represent mean *Ifnβ* expression relative to that of *Actb* ± SD (*n* = 5). ***P<0.001.

1,25(OH)_2_D_3_ reportedly inhibits osteoclast differentiation induced by M-CSF and RANKL by inhibiting c-Fos protein expression *in vitro* [10]. We found that, by western blot, c-Fos protein was induced by RANKL, and ED71 did not suppress c-Fos protein in osteoclasts as effectively as did 1,25(OH)_2_D_3_ (Fig. 2A). Although 1,25(OH)_2_D_3_ reportedly inhibits osteoclastogenesis induced by M-CSF and RANKL via *Ifnβ* induction *in vitro* [11], we found that, unlike 1,25(OH)_2_D_3_, ED71 did not induce *Ifnβ* expression in osteoclasts (Fig. 2B).

### The VDR is required for both 1,25(OH)_2_D_3_ and ED71 activity on osteoclasts

**Figure 3:**
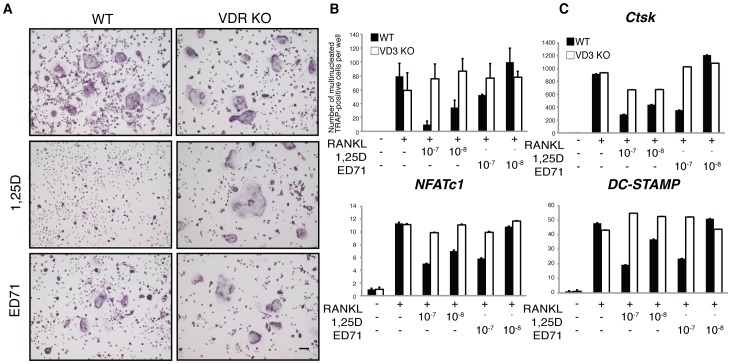
ED71 or 1,25(OH)_2_D_3_ activity requires the VDR. (**A, B** and **C**) M-CSF-dependent osteoclast progenitor cells were isolated from wild-type (WT) or VDR-deficient (VDR KO) mice and cultured in the presence of M-CSF alone (50 ng/ml) or M-CSF + RANKL (25 ng/ml) with or without indicated concentrations of ED71 or 1,25(OH)_2_D_3_ for 5 days. Cells were then stained with TRAP (**A**), and multi-nuclear TRAP-positive cells were counted (**B**). Expression of *Ctsk*, *NFATc1* and *DC-STAMP* was assessed by realtime PCR (**C**). Data represent mean *Ctsk*, *NFATc1* or *DC-STAMP* expression relative to that of *Actb* ± SD (*n* = 5).

Since 1,25(OH)_2_D_3_ and ED71 activities differ in osteoclasts, we utilized vitamin D receptor (VDR)-deficient mice to test whether both compounds act on osteoclasts via the VDR (Fig. 3). Osteoclast progenitors were isolated from wild-type and VDR-deficient mice and cultured in the presence of M-CSF and RANKL with or without 1,25(OH)_2_D_3_ or ED71 (Fig. 3). Inhibitory effects of either 1,25(OH)_2_D_3_ or ED71 on osteoclast differentiation were not seen in VDR-deficient osteoclasts (Fig. 3A and B). Similarly, inhibition of the expression of osteoclastic genes *Ctsk*, *NFATc1* and *DC-STAMP* seen following 1,25(OH)_2_D_3_ or ED71 treatment was absent in osteoclasts lacking the VDR (Fig. 3C).

**Figure 4:**
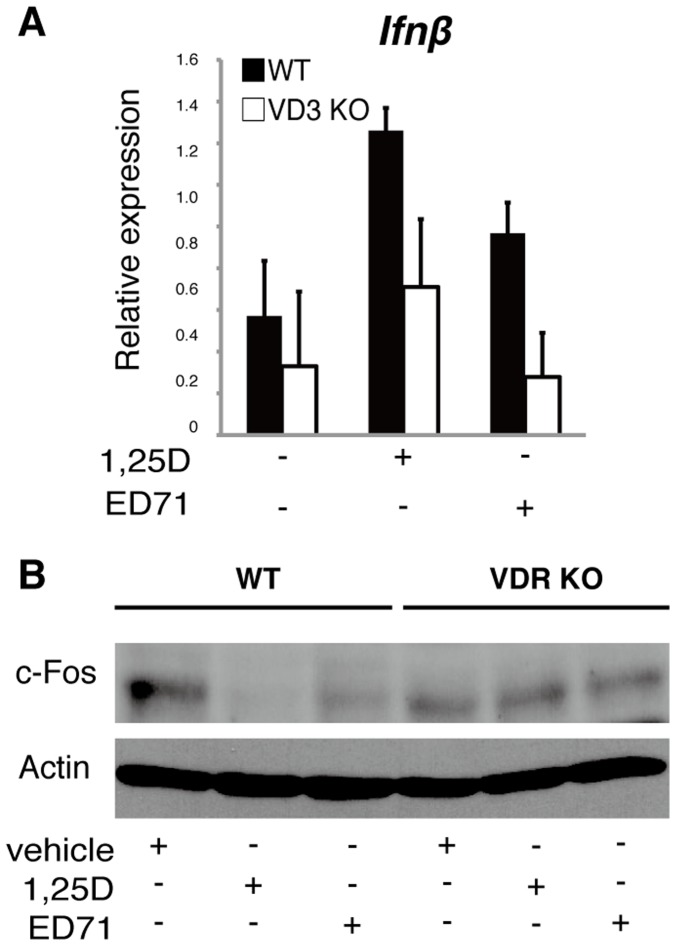
ED71 or 1,25(OH)_2_D_3_ induce *Ifnβ* and suppress c-Fos protein through the VDR. (**A** and **B**) M-CSF-dependent osteoclast progenitor cells were isolated from wild-type or VDR-deficient mice and cultured in the presence of M-CSF alone (50 ng/ml) or M−CSF + RANKL (25 ng/ml) with or without 10^−7^ M of ED71 or 1,25(OH)_2_D_3_ (1,25D) for 5 days. *Ifnβ* expression was then analyzed by realtime PCR (**A**). Data represent mean *Ifnβ* expression relative to that of *Actb* ± SD (*n* = 5). c-Fos protein was analyzed by western blot (**B**).

Moreover, decreased c-Fos protein and elevated *Ifnβ* expression seen following treatment with 1,25(OH)_2_D_3_ or ED71 were abrogated in VDR-deficient osteoclasts (Fig. 4A and B), supporting the idea that both compounds act on osteoclasts via the VDR.

### HIF1α is a target of ED71 but not 1,25(OH)_2_D_3_ in osteoclasts

**Figure 5:**
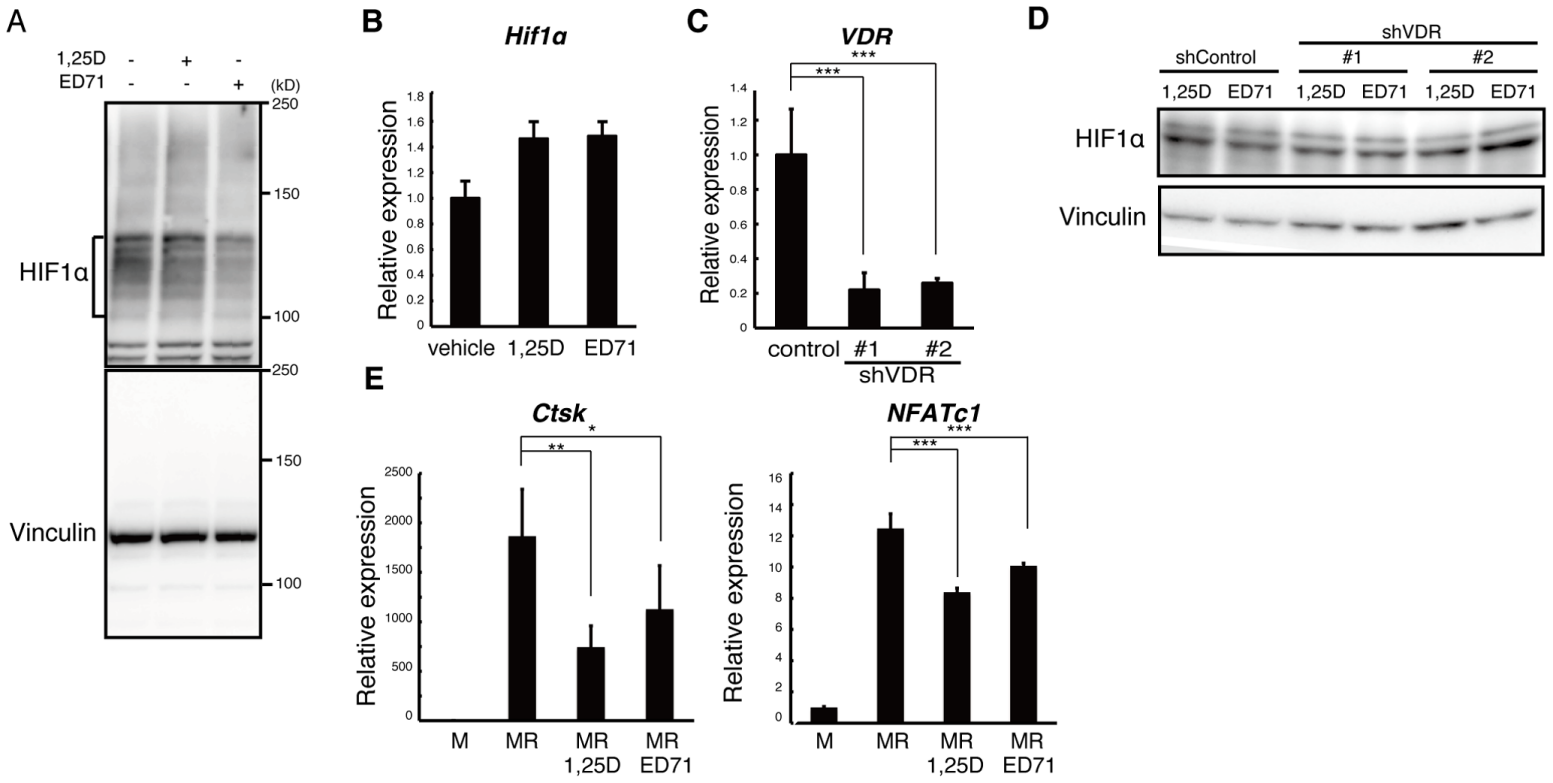
HIF1α protein is suppressed by ED71 but not by 1,25(OH)_2_D_3_. (**A**) Western analysis of Raw264.7 cells cultured in hypoxic conditions with or without 10^−7^ M of ED71 or 1,25(OH)_2_D_3_ (1,25D). (**B**) *Hif1α* mRNA levels in Raw264.7 cells cultured in hypoxic conditions were analyzed by realtime PCR in the presence or absence of 10^−7^ M ED71 or 1,25(OH)_2_D_3_. Data represent mean *Hif1α* expression relative to that of *Actb* ± SD (*n* = 5). (**C**) Levels of *VDR* transcripts in Raw264.7 cells transfected with shRNA targeting the VDR (shVDR) or control shRNA (Control) were determined by realtime PCR. Data represent mean *VDR* expression relative to that of *Actb* ± SD (*n* = 5). (**D**) Western analysis of control (shControl) or VDR-suppressed (shVDR#1 or shVDR#2) Raw264.7 transformants cultured in hypoxic conditions with ED71 or 1,25(OH)_2_D_3_ (1,25D), both at 10^−7^ M. (**E**) M-CSF-dependent Ctsk Cre/*Hif^flox/flox^* cells were cultured in normoxic conditions to suppress HIF1α in the presence of M-CSF (50 ng/ml) plus RANKL (25 ng/ml) with either ED71 or 1,25(OH)_2_D_3_ (1,25D) both at 10^−7^M for 4 days. Expression of *Ctsk* and *NFATc1* was then assessed by realtime PCR. Data represent mean *Ctsk* or *NFATc1* expression relative to that of *Actb* ± SD (*n* = 5). *P<0.05; **P<0.01; ***P<0.001.

Next, we asked whether HIF1α is a target of ED71 in osteoclasts (Fig. 5). Interestingly, we found that in cultured osteoclasts, HIF1α protein levels were suppressed by ED71 but not by 1,25(OH)_2_D_3_ (Fig. 5A). In contrast, *Hif1α* mRNA expression in osteoclasts was not inhibited by either treatment (Fig. 5B), suggesting that ED71 suppresses HIF1α at the protein level as demonstrated by estrogen treatment [14]. To determine if the VDR is required for ED71-mediated HIF1α protein suppression in osteoclasts, we generated two independent VDR knockdown Raw264.7 lines using shVDR#1 and shVDR#2 as well as a control (shControl) line (Fig. 5C) and then treated cells with ED71 or 1,25(OH)_2_D_3_ (Fig. 5D). HIF1α protein suppression by ED71 seen in control cells was abrogated in both VDR knockdown lines, suggesting that HIF1α protein suppression by ED71 is VDR-dependent. We then isolated osteoclast progenitors from Ctsk Cre/*Hif1α^flox/flox^* mice, cultured them in normoxic conditions to suppress HIF1α protein, and treated cells with or without ED71 or 1,25(OH)_2_D_3_ (Fig. 5E). ED71 treatment effectively inhibited osteoclast differentiation, even in HIF1α-suppressed cells, suggesting that ED71 likely targets factors other than HIF1α protein in osteoclasts (Fig. 5E). However, ED71 was less effective than 1,25(OH)_2_D_3_ in inhibiting osteoclastogenesis in HIF1α-suppressed cells (Fig. 5E).

## Discussion

Postmenopausal osteoporosis treatment is required to prevent disruption of daily activity or adverse outcomes due to fragile fractures. Among anti-osteoporosis agents, anti-bone resorptive or bone-forming agents include bisphosphonates, selective estrogen receptor modulator (SERM), ED71 and denosumab, or teripararide, respectively. Strong inhibition of osteoclastic activity beyond physiological levels by bisphosphonates frequently causes adverse effects such as osteonecrosis of the jaw or severely suppressed bone turnover (SSBT) [19] [20]. Meanwhile, teriparatide treatment is limited to less than two years in order to prevent development of tumors, particularly osteosarcoma.

Recently, we showed that HIF1α protein accumulation in osteoclasts following estrogen-deficiency was accompanied by osteoclast activation and bone loss in mice [14]. Either osteoclast-specific HIF1α conditional knockout or wild-type mice administered a HIF1α inhibitor were protected from OVX-induced osteoclast activation and bone loss. Moreover, HIF1α inhibition did not interfere with physiological osteoclast activities [14]. Thus, blocking HIF1α pharmacologically could represent an ideal treatment for postmenopausal osteoporosis, as it could target pathologically-activated osteoclasts without altering physiological osteoclastogenesis required for bone turnover. In this study, we found that both ED71, which is used as therapeutic agents for postmenopausal osteoporosis therapy, inhibits HIF1α protein expression. Indeed, patients treated with ED71 exhibit reduced osteoclastic activity and increased bone mass without adverse effects such as osteopetrosis [12], jaw osteonecrosis or SSBT, as seen in treated bisphosphonate-treated patients [19] [20].

Bone is a target tissue of vitamin D, and indeed, VDR was identified in osteoblasts [21]–[24]. In contrast, it is controversial whether the VDR is expressed in osteoclasts, with some authors reporting expression [25]–[28] and others not [21], [23], [29], [30]. Recently, Wang et al. demonstrated that the VDR is not expressed in multi-nuclear osteoclasts using immunohistochemistry of EGTA-decalcified adult mouse bones [21]. In addition, direct effects of 1,25(OH)_2_D_3_ have been demonstrated in osteoclasts and osteoclast progenitors [10] [11], and here we report that these effects are VDR-dependent (Fig. 3). Taken together, these studies suggest that extremely low levels of the VDR in osteoclasts may be sufficient to transduce vitamin D signals.

ED71 and 1,25(OH)_2_D_3_ have been demonstrated to inhibit osteoclast-bone resorption activity by reducing expression of the sphigosine-1-phosphate receptor 2 (S1PR2) in circulating osteoclast precursor cells and blocking the migration of these cells to the bone surface by S1P; although differences in pharmacological action between ED71 and 1,25(OH)_2_D_3_ were not demonstrated [31]. Here, we observed that, although 1,25(OH)_2_D_3_ was more potent than ED71 in inhibiting osteoclastogenesis induced by M-CSF and RANKL *in vitro*, HIF1α inhibition in osteoclasts was specific to ED71. We also found that ED71 inhibited osteoclastogenesis even in HIF1α-suppressed cells, suggesting that ED71 likely targets factors other than HIF1α protein in osteoclasts. However, ED71 was less effective than 1,25(OH)_2_D_3_ in inhibiting osteoclastogenesis in HIF1α-suppressed cells, which contrasts with observations seen in patients where the effect of ED71 on osteoclastogenesis is superior to that of 1,25(OH)_2_D_3_ [12]. The cause of this difference remains to be elucidated, but the difference of potential activity to target HIF1α-protein in osteoclasts explains, at least in part, this difference. In addition, it is possible that ED71 inhibits osteoclastogenesis through effects on different cell types. Further investigations are needed to define molecular actions of vitamin D3 analogues on bone metabolism. Nonetheless, HIF1α inhibition could serve as an index to assess osteoclastogenesis *in vitro* when developing anti-osteoporosis agents. Moreover, our study indicates that targeting HIFα could constitute an effective treatment for osteoporosis, one that would not interfere with physiological bone turnover.
